# Low antimicrobial resistance in general practice patients in Rotterdam, the city with the largest proportion of immigrants in the Netherlands

**DOI:** 10.1007/s10096-019-03804-8

**Published:** 2020-01-06

**Authors:** Maaike Honsbeek, Aimée Tjon-A-Tsien, Ellen Stobberingh, Jurriaan de Steenwinkel, Damian C. Melles, Jan Lous, Jan Hendrik Richardus, Hélène Voeten

**Affiliations:** 1 Municipal Public Health Service Rotterdam-Rijnmond, Schiedamsedijk 95, 3011 EN Rotterdam, The Netherlands; 2 Department of Health Care, University of Applied Sciences Leiden, Leiden, The Netherlands; 3 Department of Medical Microbiology, CAPHRI, Maastricht University, Maastricht, The Netherlands; 4 Department of Medical Microbiology and Infectious Diseases, Erasmus MC, University Medical Center Rotterdam, Rotterdam, The Netherlands; 5 Department of Medical Microbiology, Meander Medical Centre, Amersfoort, the Netherlands; 6 Medical Laboratory for Primary Care, Star-SHL, Rotterdam, The Netherlands; 7 Department of Public Health, Erasmus MC, University Medical Center Rotterdam, Rotterdam, The Netherlands

**Keywords:** Antimicrobial resistance, Prevalence, General practice

## Abstract

Antimicrobial resistance (AMR) is an increasing problem. The prevalence of antimicrobial resistance in general practice patients is expected to be relatively high in Rotterdam, the Dutch city with the largest proportion non-Western immigrants. The aim of this study was to assess the prevalence of antibiotic-resistant uropathogens (*Escherichia coli*, *Klebsiella pneumoniae*, and *Proteus mirabilis*) in general practices in Rotterdam, and to find a possible association between the prevalence of antibiotic-resistant *E. coli* and age, gender, and socioeconomic status (SES). A retrospective analysis was performed of urine samples from general practice patients in 2016. The prevalence of AMR in uropathogens was compared with national resistance data, as was the prevalence of highly and multidrug resistant and extended spectrum β-lactamase (ESBL) producing *E. coli* and *K. pneumoniae.* Univariate logistic regression was used to study associations between antibiotic-resistant *E. coli* and age, gender, and SES area score. No clinically relevant differences were observed in the prevalence of antibiotic-resistant uropathogens in Rotterdam compared with the national prevalence. For *E. coli* and *K. pneumoniae*, the prevalence was 3.6% for ESBL production (both pathogens together), while the prevalence ranged between 4.2%–5.0% for high resistance and between 1.2%–3.3% for multidrug resistance. Ciprofloxacin-resistant *E. coli* was significantly associated with higher age. Although Rotterdam has a high percentage of non-western immigrants and a low SES, AMR is low among general practice patients. This indicates that adherence to national guidelines in general practice enables maintenance of low AMR, even in high-risk populations.

## Introduction

Antimicrobial resistance (AMR) is an increasing public health problem. Although the prevalence of antibiotic-resistant microorganisms in the Netherlands is low compared with other countries in Europe and worldwide, resistance is increasing [1]. National data on the prevalence of antibiotic-resistant microorganisms are published annually in NethMap [2]. The data are obtained from the Infectious Diseases Surveillance Information System-Antimicrobial Resistance (ISIS-AR), a national surveillance system among microbiological laboratories that send their data—which are routinely obtained from patient samples—for analysis to the National Institute of Public Health and the Environment (RIVM) [2]. The results from urine samples from general practice (GP) patients with (putative) urinary tract infections (UTI) are also included in NethMap. These samples are mainly from patients after treatment failure of initial therapy according to the National Guidelines (NHG) [2, 3]. Risk factors contributing to the carriage of or infection with an antibiotic-resistant microorganism in the community include travel to foreign countries, frequent use of antibiotics, being born outside the Netherlands, recent (foreign) hospitalization, higher age, and comorbidity [4–8].

The population of Rotterdam—the second largest city in the Netherlands—consists of 38% non-Western immigrants, mostly from Suriname, Turkey, Morocco, and other countries [9]. They may travel often to their home country to visit family and friends and may have an increased risk of coming into contact with antibiotic-resistant microorganisms. Furthermore, they have a higher morbidity (22% more loss of healthy life years due to morbidity than the native Dutch population), which can lead to frequent use of antibiotics, one of the main drivers of AMR [10]. Besides having the highest proportion of non-Western immigrants, Rotterdam on average has a lower socioeconomic status (SES) than the Netherlands in general [11]. A low individual SES can be considered an indicator of poor immune regulation and impoverished health condition, and thus a risk factor for AMR [12, 13]. In addition, patients with antibiotic-resistant infections are more likely to be unemployed, to have a lower educational attainment, and a lower income [14]. Due to the high proportion of immigrants and the low SES score, it could be expected that the prevalence of AMR in the Rotterdam population is higher than the national AMR prevalence reported in NethMap.

Until 2017, no data were publicly available on the prevalence of antibiotic-resistant microorganisms among GP patients in Rotterdam. The objectives of this study were firstly to establish the prevalence of antibiotic-resistant uropathogens from GP patients in Rotterdam, i.e., *Escherichia coli* (*E. coli*), *Klebsiella pneumoniae* (*K. pneumoniae*), and *Proteus mirabilis* (*P. mirabilis*), and the prevalence of highly and multidrug resistant and extended spectrum β-lactamase (ESBL)–producing *E. coli* and *K. pneumoniae*. Secondly, we aimed to assess the association between the prevalence of antibiotic-resistant *E. coli* and age, gender and SES score. We hypothesised that the prevalence of antibiotic resistance in Rotterdam is higher than the national prevalence of AMR, due to its higher proportion of non-Western immigrants and the lower SES score.

## Materials and methods

### Design and procedures

We conducted a retrospective analysis of microbiological results from the GP laboratory (Star-SHL) of urine samples submitted in 2016. The dataset consisted of the four-digit postal codes of both the GP and the patient, an encrypted unique patient code, year of birth and gender of the patient*,* date the urinary samples were received, and the microbiological culture results. The culture results included the following: the isolated micro-organism, the antibiotic susceptibility, and the presence of ESBL.

A urine culture was considered positive when > 10e3 colony forming units per ml of one bacterial species were detected. The presence of ≥ 2 bacterial species was considered mixed flora and was not included in the analyses. Antibiotic susceptibility was tested using the VITEK® 2 COMPACT system (Biomerieux, Lyon) and interpreted according to the EUCAST guidelines of 2016 [15]. The resistance percentages of the most common uropathogens, i.e., *E. coli*, *K. pneumoniae*, and *P. mirabilis* were determined for amoxicillin, co-amoxiclav, cefuroxime, cefotaxime, ceftazidime, ciprofloxacin, gentamicin, tobramycin, fosfomycin, trimethoprim, co-trimoxazole, and nitrofurantoin. Aminoglycosides (gentamicin and tobramycin) were only included for comparative purposes with NethMap. Because we did not have access to the underlying data of NethMap, we could not calculate statistical significance of differences between the prevalence of the Rotterdam region and the national prevalence reported in NethMap. Therefore, we applied the term that NethMap uses to compare trends over time, clinically relevant differences [2]. This is defined by NethMap as a difference of ≥ 5% for prevalences ≥ 10%, and of ≥ 2.5% for prevalences < 10%. A difference in resistance between Star-SHL data and the NethMap data was considered clinically relevant according to this definition, with the NethMap prevalence data serving as reference [2]. One isolate per patient per uropathogen, and only the first isolate in 2016, was included in the final analysis to avoid bias of repeated isolates of a patient. The production of ESBL and the prevalence of highly resistant microorganisms (HRMO) and multidrug resistance in *E. coli* and *K. pneumoniae* were determined according to the criteria used in NethMap [2]. ESBL production was determined as resistant to cefotaxime/ceftriaxone and/or ceftazidime as indicator agents. HRMO was defined as ESBL positive, or resistant to both fluoroquinolones (ciprofloxacin) and aminoglycosides (gentamicin or tobramycin). Multidrug resistance was defined as resistant to co-amoxiclav and ciprofloxacin and co-trimoxazole. Because *E. coli* and *K. pneumoniae* are the most common Enterobacteriaceae for determining ESBL production, HRMO, and multidrug resistance, we used these in our study. We excluded the prevalence of resistance in mixed specimens, due to the absence of sensitivity data in these mixed specimens.

## Statistical methods

We calculated the associations between antibiotic-resistant *E. coli* and age, gender, and SES area score using univariate logistic regression analysis*.* We used the prevalence of antibiotic-resistant *E. coli* as the outcome variable in the regression analyses because it was the most common uropathogen. Age was calculated in years (2016 minus the year of birth). We included age as a dichotomous variable (< 70 years and ≥ 70 years). We analysed gender as a binary outcome variable (male/female). Because individual SES was unknown, we used SES score at the neighbourhood level (‘SES area score’) as a proxy for the individual SES. SES area score is calculated by the Social and Cultural Planning Office (SCP) and is based on the average income and the percentage of low-educated people, unemployed people, and people with a low income per neighbourhood/postal code. According to the SCP, a SES area score of less than − 1 is a low score, a score between − 1 and + 1 is average, and a score of more than 1 is considered as high [16]. Only the postal codes 3000–3089 of the city of Rotterdam were included in our analyses. We considered *p* values < 0.05 as statistically significant. To reduce the risk of a type I error, we adjusted the *p* values for multiple testing using the Holm-Bonferroni method. Statistical descriptive analyses and univariate logistic regression were performed using the data.table-, glm-, and the dplyr function in RStudio 1.1.383 [17].

## Results

In 2016, 7966 urine samples were submitted to Star-SHL from patients from Rotterdam. Of these, 21.8% (*n* = 1734) were culture positive, and 2.7% (*n* = 218) were mixed flora. The median age of the patients was higher in men (75 years) than in women (69 years), and the male/female ratio was 20:80. More than 5000 patients (65.6%, *n* = 5226) had a low SES area score, 27.0% (*n* = 2151) and 7.4% (*n* = 589) had an average and high SES area score respectively.

The age of patients with positive cultures ranged from 15 to 103 years, of whom 50.0% belonged to the age group of ≥ 70 years. *E. coli* was the most common uropathogen (*n* = 1130; 65%), followed by *K. pneumonia* (*n* = 167; 10%) and *P. mirabilis* (*n* = 120; 7%). The remaining 18% (*n* = 317) included 54 other species, such as *Pseudomonas aeruginosa, Enterococcus* spp*.*, and *Klebsiella oxytoca.* Most *E. coli*–positive samples were from patients with a low SES area score (*n* = 701, 62.0%) followed by a middle SES area score (*n* = 346; 30.6%) and high SES area score (*n* = 83; 7.4%).

The highest prevalence of resistance among *E. coli* was to amoxicillin (41.0%), among *K. pneumoniae* to fosfomycin (32.0%), and among *P. mirabilis* to trimethoprim (31.0%) (Table 1). There were no clinically relevant differences in the prevalence of resistance to the antibiotics tested between isolates from Rotterdam and the national data, except for tobramycin in *P. mirabilis* that was higher in Rotterdam (7.0%) than in NethMap (3.0%). The prevalence of ESBL producing *E. coli* and *K. pneumoniae* together in Rotterdam was 3.6% and comparable with the national prevalence of 3.1% for all Enterobacteriaceae (Table 2)*.* The prevalence of HRMO was 5.0% in *E. coli* and 4.2% in *K. pneumoniae* compared with both 5.0% in national data. Multidrug resistance was 3.3% in *E. coli* and 1.2% in *K. pneumoniae,* compared with a national prevalence of 3.0% and 2.0%, respectively.

**Table 1:** Prevalence of antimicrobial resistance for three different uropathogens compared with the national prevalence in 2016

Median age	*E. coli*		*K. pneumoniae*		***P. mirabilis***	
	Rotterdam (*n* = 1130)	Netherlands (*n* = 57,948)^a^	Rotterdam (*n* = 167)	Netherlands (*n* = 7801)^a^	**Rotterdam** (*n* = 120)	**Netherlands** (*n* = 5967)^a^
	69 years	66 years	74 years	73 years	79 years	75 years
Antibiotic						
Amoxicillin	41%	39%	–	–	19%	21%
Co-amoxiclav	17%	20%	11%	10%	5%	5%
Cefuroxime	9%	7%	14%	14%	2%	1%
Cefotaxime	3%	3%	5%	5%	1%	1%
Ceftazidime	2%	2%	5%	4%	0%	0%
Ciprofloxacin	11%	9%	4%	4%	6%	7%
Gentamicin	3%	4%	4%	2%	7%	5%
Tobramycin	3%	4%	4%	3%	*7%*^*b*^	*3%*^*b*^
Fosfomycin	2%	1%	32%	32%	20%	17%
Trimethoprim	26%	25%	20%	22%	31%	35%
Co-trimoxazole	25%	23%	11%	11%	28%	28%
Nitrofurantoin	2%	2%	–	–	–	–

^a^Age > 12 years

^b^Italicized reported figures: the difference in resistant *P. mirabilis* against tobramycin between Star-SHL and NethMap is clinically relevant; i.e. absolute difference of ≥ 2.5% for prevalences < 10%

**Table 2:** Prevalence of ESBL, HRMO and multidrug resistance compared with the national prevalence in 2016

	Rotterdam		Netherlands	
	***E. coli*+*K. pneumonia*together (n = 1297)**		**All Enterobacteriaceae (*n* = 101,330)**	
ESBL^a^	3.6%^b^		3.1%^c^	
	***E. coli*(*n* = 1130)**	***K. pneumoniae*(*n* = 167)**	***E. coli*(*n* = 57,948)**^d^	***K. pneumoniae*(*n* = 7801)**^d^
HRMO^e^	5.0%	4.2%	5.0%	5.0%
Multidrug resistance^f^	3.3%	1.2%	3.0%	2.0%

*ESBL* Extended spectrum beta-lactamase

*HRMO* Highly Resistant Microorganisms

^a^ESBL was determined as resistant to cefotaxime (or ceftriaxone) and/or ceftazidime

^b^Determined for *E. coli* and *K. pneumonia* together

^c^Determined for all Enterobacteriaceae

^d^Age > 12 years

^e^HRMO was defined as resistant to cefotaxime/ceftriaxone and/or ceftazidime as indicator agents for the production of ESBL, or resistant to both fluoroquinolones (ciprofloxacin) and aminoglycosides (gentamycin or tobramycin) in *E. coli* and *K. pneumonia*

^f^Multidrug resistance was defined as resistant to co-amoxiclav and ciprofloxacin and co-trimoxazole in *E. coli* and *K. pneumoniae*

When age was dichotomised (< 70 years and ≥ 70 years), higher age was associated with ciprofloxacin and nitrofurantoin resistant *E. coli* (OR 1.86; 95% CI 1.27–2.73; *p* = 0.001 and OR 4.11; 95% CI 1.50–14.41; *p* = 0.01, respectively) (Table 3). However, after adjustment for multiple testing using the Holm-Bonferroni method, higher age was only significantly associated with ciprofloxacin resistance (Bonferroni corrected *p* = 0.03). A low SES area score was associated with higher resistance to cefuroxime (OR 4.93; 95% CI 1.51–30.35; *p* = 0.03), but after adjustment for multiple testing, this effect was not statistically significant. Gender was not associated with antibiotic resistant *E. coli*.

**Table 3:** Factors associated with resistant *E. coli* in 2016 (*n* = 1130) using univariate logistic regression analysis

	Age ≥ 70 years versus < 70 years			Female vs. male			Low SES score vs. high SES score			Middle SES score vs. high SES score		
Antibiotic	OR	95% CI	*p*	OR	95% CI	*p*	OR	95% CI	*p*	OR	95% CI	*p*
Nitrofurantoin^a^	4.11*	1.50–14.41	0.01	1.00	0.33–4.30	1.00	NA^b^	NA^b^	NA^b^	NA^b^	NA^b^	NA^b^
Fosfomycin^a^	2.34	0.99–6.12	0.06	3.94	0.82–70.73	0.18	1.92	0.38–34.79	0.53	1.45	0.24–27.53	0.73
Trimethoprim^a^	1.20	0.92–1.57	0.18	1.13	0.78–1.68	0.52	0.77	0.47–1.29	0.31	0.87	0.52–1.48	0.59
Co-trimoxazole	1.10	0.83–1.44	0.51	1.22	0.83–1.84	0.32	0.82	0.50–1.39	0.45	0.85	0.50–1.48	0.55
Amoxicillin	1.12	0.88–1.41	0.36	0.94	0.68–1.31	0.71	0.77	0.49–1.22	0.26	0.78	0.48–1.26	0.30
Co-amoxiclav	1.14	0.84–1.54	0.41	0.88	0.59–1.35	0.54	1.19	0.66–2.31	0.56	1.11	0.59–2.21	0.76
Cefuroxime	1.15	0.76–1.75	0.51	1.14	0.64–2.18	0.68	4.93*	1.51 – 30.35	0.03	2.62	0.75–16.57	0.20
Ciprofloxacin	1.86*	1.27–2.73	0.001^c^	0.69	0.44 - 1.13	0.13	1.48	0.70–3.62	0.34	0.99	0.44–2.54	0.99

*SES* social economic status

*Statistically significant associations

^a^These antibiotics should be prescribed in this order (first, second and third choice) by the GPs according to the National Guideline for UTI [23]

^b^*NA* not applicable: too few outcome events (high SES score has 0 resistant *E. coli* to nitrofurantoin)

^c^Significant after adjustment for multiple testing using the Holm-Bonferroni method, *p* = 0.03

## Discussion

This study provides insight into the prevalence of AMR of *E. coli*, *K. pneumoniae*, and *P. mirabilis* in general practice in Rotterdam. The prevalence of AMR among these microorganisms was in general comparable with the national data in NethMap 2016. Higher age was significantly associated with *E. coli* resistance for ciprofloxacin. We found no association between gender or SES area score and resistant *E. coli*.

We hypothesised that the prevalence of antibiotic resistance in Rotterdam would be higher than the national prevalence of AMR in 2016, due to the higher proportion of non-Western immigrants and the lower SES area score. However, our results do not support this hypothesis. The reasons for the rejection of our hypothesis remain unclear. Furthermore, we found no association between AMR and SES. Perhaps the use of SES area scores instead of individual SES scores underestimates a possible association. With area scores, individual differences in SES are levelled out. In addition, we have no data about migration status, travel behaviour, and other individual socioeconomic factors. Pini et al. [14] speculated that there are several pathways of lower socioeconomic conditions that could lead to a higher occurrence of infectious diseases, such as affected living conditions due to poor housing, overcrowding, poor nutrition, unsatisfactory hygiene, unhealthy lifestyle, scarce health education and knowledge, and reduced immunisation. We would need to link laboratorial data to individual migration and socioeconomic factors to further explore the association between AMR and possible risk factors.

A strength of our study is that it includes many positive urine cultures (more than 1700). It is the first study that presents data on the prevalence of AMR and the associations between resistance and risk factors age, gender, and SES area score in GP patients in Rotterdam. A limitation is that we only included data from GPs who are affiliated with the Star-SHL laboratory, which is estimated to be approximately 65% of all GPs in Rotterdam. We have no information on the number of urine samples that were examined in this laboratory compared with the total number of patients who visited their GP with UTI complaints. The resistance data obtained are most likely from patients not responding to the first (and possibly second) empiric therapy choice nitrofurantoin, fosfomycin, and trimethoprim, which is comparable with NethMap. However, these patients are not representative of the resistance in “unselected” samples of GP patients with a UTI.

The prevalence of antibiotic-resistant uropathogens in Rotterdam was comparable with the national data. The only difference, namely tobramycin-resistant *P. mirabilis* (7.0% versus 3.0%), can be explained by different cutoff values. The NethMap data are based on the EUCAST guidelines, i.e. a cutoff MIC value > 4 mg/L for resistance of Enterobacteriaceae to aminoglycosides including tobramycin. STAR-SHL used cutoff values of > 2 mg/L as resistance criterion due to the phenotype interpretation of the expert system of VITEK, resulting in a higher resistance prevalence than NethMap.

The prevalence of ESBL among *E. coli* and *K. pneumoniae* in our study (3.6%) was comparable with the national data. Other studies reported prevalences ranging from 4.5 to 19.1% [18–25]. It should be noted, however, that these other studies have included populations that are not comparable with our patient population*.* Van der Bunt et al. [18] and Koningstein et al. [19] included children and adults at day-care centres and also included *Enterobacter cloacae*; Wielders et al. [20] and Huijbers et al. [21] investigated a population in areas with many livestock and broilers, where Wielders included *Enterobacter* spp., *Citrobacter freundii*, *Morganelli morganii*, and *Hafnia alvei* for further analysis if they displayed an ESBL phenotype. Reuland et al. [22, 23] included patients with and without gastrointestinal complaints and included one *Shigella sonnei* isolate. In these studies, faecal samples were examined. Paltansing et al. [24] included travellers and rectal swabs, whereof one isolate was *Citrobacter freundii*, and Platteel et al. [25] analysed perianal and faeces swabs from patients admitted to the hospital. The study population of Star-SHL is most comparable with the population of NethMap, i.e. urine samples of patients from GPs submitted mostly after treatment failure. However, we only included *E. coli* and *K. pneumoniae* to determine the prevalence of ESBL, whereas NethMap’s ESBL prevalence was based on isolates of all Enterobacteriaceae. One would expect a higher ESBL-prevalence when including all Enterobacteriaceae, but this was not the case.

Nitrofurantoin is the first choice for treating UTI according to the NHG guidelines [3]. Although the prevalence of nitrofurantoin resistance was low in Rotterdam and at the national level, we found that resistance in the age group of ≥ 70 years was higher than in the group < 70 years (OR 4.11; 95% CI 1.50–14.41; *p* = 0.01). After correction for multiple testing, this association was not statistically significant, but an impaired kidney function, which often occurs in the elderly, can make nitrofurantoin less effective in this age group [26]. Therefore, caution is needed when prescribing nitrofurantoin to older patients.

Females are known to be more prone to UTIs than males [27, 28]. A UTI in females is usually uncomplicated without tissue invasion, whereas in males it is more often complicated because of prostate involvement [3]. Treatment for an uncomplicated UTI includes agents that give high concentrations in the urine, such as nitrofurantoin and fosfomycin. For males, the agents need to reach a sufficiently high concentration in the prostate, such as co-trimoxazole and ciprofloxacin. Although our study sample contained more women than men, there were enough men in our analysis when comparing resistance in men versus women (20.0%, *n* = 347). We found no differences in the prevalence of resistance of these last two agents between males and females and therefore concluded that gender was not a risk factor for resistance in our study.

In conclusion, the prevalence of AMR in the Rotterdam region is low. This indicates that adherence to national guidelines in general practice enables maintenance of low AMR, even in high-risk populations. To explore the associations between migration, socioeconomic factors, and AMR, these variables need to be included in further studies.
